# Association between the dual use of electronic and conventional cigarettes and NAFLD status in Korean men

**DOI:** 10.18332/tid/159167

**Published:** 2023-02-24

**Authors:** Minjung Han, Seogsong Jeong, Jihun Song, Sun Jae Park, Cheol Min Lee, Kiheon Lee, Sang Min Park

**Affiliations:** 1 Department of Family Medicine, Myongji Hospital, Goyang, Republic of Korea; 2 Department of Biomedical Informatics, CHA University School of Medicine, CHA University, Seongnam, Republic of Korea; 3 Institute for Biomedical Informatics, School of Medicine, CHA University, Seongnam, Republic of Korea; 4 Department of Biomedical Sciences, Seoul National University Graduate School, Seoul, Republic of Korea; 5 Department of Family Medicine, Healthcare System Gangnam Center, Seoul National University Hospital, Seoul, Republic of Korea; 6 Department of Family Medicine, Seoul National University College of Medicine, Seoul, Republic of Korea; 7 Department of Family Medicine, Seoul National University Bundang Hospital, Seongnam, Republic of Korea; 8 Department of Family Medicine, Seoul National University Hospital, Seoul, Republic of Korea

**Keywords:** smoking, non-alcoholic fatty liver disease, dual use, electronic inhalable products, scoring systems

## Abstract

**INTRODUCTION:**

This study investigated the association between smoking types, including dual use (usage of both combustible cigarettes and e-cigarettes), and non-alcoholic fatty liver disease (NAFLD) status in Korean men.

**METHODS:**

Data from the 7th and 8th Korea National Health and Nutrition Examination Survey (KNHANES) 2016–2020 were used. The presence of NAFLD was defined by the respective cut-off values for the Hepatic Steatosis Index (HSI), NAFLD Ridge Score (NRS), and Korea National Health and Nutrition Examination Survey NAFLD score (KNS). Multivariate logistic regression analyses were used to determine the associations between smoking types and NAFLD as determined by HSI, NRS, and KNS.

**RESULTS:**

After adjustment for confounders, an independent association was observed between dual use and NAFLD (HSI: AOR=1.47; 95% CI: 1.08–1.99, p=0.014; NRS: AOR=2.21; 95% CI: 1.70–2.86, p=0.000; KNS: AOR=1.35; 95% CI: 1.01–1.81, p=0.045). Cigarette only smokers also had significantly higher odds of NAFLD compared to never smokers for all of the NAFLD indices (HSI: AOR=1.22; 95% CI: 1.05–1.42, p=0.008; NRS: AOR=2.13; 95% CI: 1.87–2.42, p=0.000; KNS: AOR=1.33; 95% CI: 1.14–1.55, p=0.000). In subgroup analyses, no significant interaction effects were found for age, BMI, alcohol consumption, income, physical activity, and the diagnosis of T2DM. Moreover, cigarette only smokers and dual users differed significantly in terms of log-transformed urine cotinine and pack-years. The relationship between smoking type and pack-years was attenuated after stratification by age.

**CONCLUSIONS:**

This study shows that the dual use of e-cigarettes and combustible cigarettes is associated with NAFLD. Age differences may explain why dual users, with a greater proportion of young people, appear to have fewer pack-years than cigarette only smokers. Further research should be conducted to investigate the adverse effects of dual use on hepatic steatosis.

## INTRODUCTION

Smoking is a major source of preventable illness and mortality around the globe. Although smoking rates are declining due to global tobacco control efforts, they remain relatively high in certain parts of the world^1,2^. Also, novel electronic inhalable products, such as nicotine vaping products (NVPs or e-cigarettes) and heated tobacco products (HTPs), are gaining popularity, especially among the younger population^3^. These novel products allow users to take in nicotine without burning tobacco^4^. While many studies have explored the effects of smoking on the cardiovascular and respiratory system, few have investigated the influence of smoking on the liver, specifically in terms of non-alcoholic fatty liver disease (NAFLD)^5^. Moreover, even fewer studies have examined the association between the dual use of combustible cigarettes and electronic inhalable products and NAFLD.

Non-alcoholic fatty liver disease (NAFLD) is an important health problem that has a worldwide prevalence of approximately 25%^6^. NAFLD consists of a range of diseases, from hepatic steatosis to non-alcoholic steatohepatitis (NASH)^7^. It is defined as the presence of excessive lipid accumulation in the liver despite the lack of alcohol consumption or other secondary causes^8^. The prevalence of NASH is approximately 6% in the general population, and 15–20% patients with NASH eventually develop cirrhosis^5^. NAFLD’s complexity and multifactorial nature make its pathogenesis challenging to understand^7^. Various genetic, environmental, and metabolic factors are hypothesized to play a role in the development of NAFLD^7^. In particular, components of the metabolic syndrome, including obesity, systemic hypertension, dyslipidemia, and insulin resistance, show a strong association with NAFLD^9^.

While biopsy remains the gold standard to diagnose NAFLD/NASH, it is invasive, costly, and has high complication rates^10^. NAFLD scoring systems may provide a more accessible way to determine the presence of fatty liver based on formulas that utilize biochemical markers, physical measurements, and the presence of underlying chronic diseases^9^. NAFLD scores contain different parameters that may reflect the distinct physiological mechanisms by which smoking leads to NAFLD. We used three different indices to assess the presence of NAFLD: the Hepatic Steatosis Index (HSI), NAFLD Ridge Score (NRS), and Korea National Health and Nutrition Examination Survey NAFLD Score (KNS). This study aimed to investigate the association between dual use and NAFLD status, defined by the respective cut-off values for the three aforementioned NAFLD scores (HSI, NRS, and KNS)^11-13^.

## METHODS

### Study population and data collection

**Figure 1:**
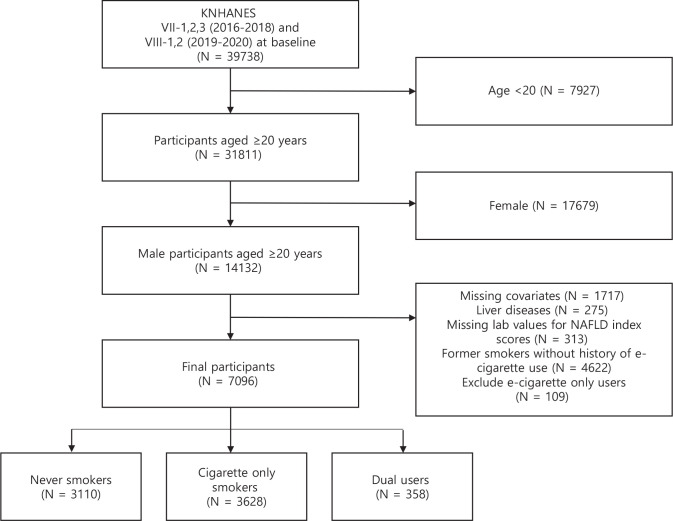
Flow diagram of the study participant selection process

This study used data from the 7th and 8th Korea National Health and Nutrition Examination Survey (KNHANES VII-1,2,3 and VIII-1,2), conducted by the Korea Center for Disease Control and Prevention. A two-stage stratified cluster sampling design was used to survey the population of South Korea in the period 2016–2020. Among the 39738 participants, we excluded participants who were aged <20 years (n=7927). Female participants were subsequently excluded, given the low rates and underreporting of smoking in this subpopulation (n=17679)^14,15^. We also excluded those who had missing covariates (n=1717), underlying chronic liver diseases such as hepatitis B, C virus infections and liver cancers (n=275), and missing laboratory values to calculate NAFLD index scores (n=313). Furthermore, former smokers without history of e-cigarette use were excluded to account for the confounding effects of smoking cessation (n=4622)^16^. Finally, we excluded e-cigarette only users due to insufficient sample size (n=109). Thus, our sample was restricted to never smokers, cigarette only smokers, and dual users. A total of 7096 participants who met these inclusion criteria were selected for this study (Figure 1).

### Assessment of NAFLD index scores

NAFLD was assessed using three previously validated fatty liver indices: Hepatic Steatosis Index (HSI), NAFLD Ridge Score (NRS), and Korea National Health and Nutrition Examination Survey Non-alcoholic Fatty Liver Disease Score (KNS)^11-13^. These indices are used for NAFLD screening purposes in the general population^17^. Previous studies have demonstrated these indices to have acceptable diagnostic accuracy for NAFLD^12,17^. The dual cut-off values for each index is used to rule in and rule out NAFLD, respectively. None of the scores can be used to classify the degree of hepatic steatosis^17^.

The derivation and validation of HSI was performed using a large cohort of >10000 subjects who participated in health checkups^11^. The score includes laboratory parameters, such as aspartate aminotransferase (AST) and alanine transaminase (ALT), BMI, gender, and the presence of type 2 diabetes (T2DM) (Supplementary file Table S1). NAFLD as determined by ultrasonography was used as the reference standard, and AUROC was 0.81^11^. At values <30.0 and >36.0, HSI excluded and detected NAFLD with a sensitivity of 93.1% and a specificity of 92.4%, respectively^11^.

NRS was calculated using a machine learning-based model that includes laboratory parameters [ALT, high-density lipoprotein (HDL), triglyceride (TG), hemoglobin A1c (HbA1c), and white blood cell (WBC) count] and the presence of hypertension (Supplementary file Table S1)^13^. NAFLD diagnosed by proton magnetic resonance spectroscopy was used as the reference standard, and AUROC was 0.87^17^. At values <0.24 and >0.44, NRS excluded and detected NAFLD with a sensitivity of 92% and a specificity of 90%, respectively^13^.

KNS was derived using 2008–2010 KNHANES data using the NAFLD liver fat score as reference without external validation^12^. It includes the following parameters: sex, waist circumference, systolic blood pressure (SBP), fasting serum glucose, triglyceride (TG), and ALT (Supplementary file Table S1). The AUROC was 0.929. The dual cut-off values for KNS were -3.285 and 0.884^12^.

### Classification of smoking status

Self-report was used in the classification of smoking type. Combustible cigarette users were defined as those who smoked more than 100 cigarettes in their lifetime and were currently smoking every day or sometimes. E-cigarette users were defined as those who responded ‘yes’ to both of the following questions: 1) ‘Have you ever used an e-cigarette in your lifetime?’, and 2) ‘Have you used an e-cigarette in the past 30 days?’. Dual users were defined as those that satisfied the criteria for both combustible cigarette and e-cigarette use. Exclusive users of either combustible cigarettes or e-cigarettes were defined as those who satisfied only one of the criteria for combustible cigarette or e-cigarette use. Never smokers were defined as those who did not satisfy any of the criteria for combustible cigarette or e-cigarette use, which signified that they have never smoked or smoked fewer than 100 cigarettes in their lifetime and were never users of e-cigarettes.

For cigarette only smokers and dual users, subtracting the age of smoking initiation from the current age yielded the duration of smoking. Pack-years of smoking were calculated by multiplying the above duration by the average number of cigarettes smoked per day.

### Key variables

*Categorial variables*

Independent variables included age (<50 years, ≥50 years), body mass index status [underweight or normal (<23.0 kg/m^2^), overweight or obese (≥23.0 kg/m^2^)], household income (lower half, upper half), education (≤9 years, >9 years); employment status (unemployed, employed), alcohol consumption (≤1 time/week, >1 time/week), physical activity (inadequate, adequate), hypertension, dyslipidemia, and T2DM status. BMI status was classified according to the Korean Society of the Study of Obesity guidelines^18^. Those who were physically active were defined as those who reported more than 150 min/week of moderate intensity activity, more than 75 min/week of high-intensity activity, or a combination of both^19^.

*Continuous variables*

For KNHANES VII and VIII (2016–2020), urine cotinine (ng/mL) was assessed in all subjects aged >6 years. It was measured by high-performance liquid chromatography – mass spectrometry (HPLC-MS) using API 4000 with an Agilent 1100 Series (AB Sciex, Framingham, MA, USA). The limit of detection for urine cotinine was 0.27 ng/mL. Since urine cotinine is not normally distributed, it was expressed as a geometric mean (95% CI) and log-transformed for analysis.

### Statistical analysis

In the descriptive analysis, the chi-squared test was used as a test of homogeneity for independent categorical variables by smoking type. Also, one-way analysis of variance (ANOVA) was used to test for significant differences in the means of NAFLD scores (continuous) between never smokers, cigarette only smokers, and dual users.

Subsequently, multivariate logistic regression analysis was conducted to estimate the adjusted odds ratio (AOR) and 95% confidence interval (CI) of NAFLD, defined by the respective cut-off values of HSI, NRS, and KNS, according to smoking type, with never smokers as reference. The cut-off values for NAFLD indices were derived in previous studies^12,13^. Adjusted odds ratios were initially calculated following adjustments for age (continuous), income (categorical), education level (categorical), and occupation (categorical) in Model 1. Model 2 was additionally adjusted for the categorial variables hypertension, T2DM, and dyslipidemia. Model 3 was further adjusted for the categorial variables BMI status, alcohol consumption, and physical activity. In addition, subgroup analyses were conducted by age, BMI, alcohol consumption, income, physical activity, and T2DM. The Wald test was used to determine whether modifiers had a significant effect on the association between smoking type and NAFLD. In supplementary analyses, independent samples t-tests using log-transformed urine cotinine and pack-years of cigarette smoking were conducted to analyze for differences between cigarette only smokers and dual users. In addition, independent samples t-tests were conducted to assess whether a significant difference in pack-years exists between cigarette only smokers and dual users in those aged <50 years and in those ≥50 years. Finally, sensitivity analyses were performed using a multivariate logistic model adjusted for logtransformed urine cotinine in addition to the existing covariates.

KNHANES is based on a complex survey design, and sampling weights were provided to account for discrepancies between the sample and the reference population. These sampling weights were used in all statistical analyses in this study; 95% confidence intervals were applied and a p<0.05 was considered statistically significant. All statistical analyses were performed using STATA ver. 14.0 (STATA Corp., College Station, TX, USA).

### Ethics statement

The Institutional Review Board of Myongji Hospital (IRB number: 2022-05-030) approved the study protocol. The ethics committee waived the need for participant consent, because the study involved routinely collected medical data that were anonymized at all stages, including during the data cleaning and statistical analysis. The methods were carried out in accordance with the relevant guidelines and regulations.

## RESULTS

Table 1 presents the baseline characteristics of the study population. In the sample of 7096 participants, 3110 were classified as never smokers, 3628 as cigarette only smokers, and 358 as dual users. The differences in age, BMI status, household income, education level, employment, alcohol consumption, physical activity, hypertension, dyslipidemia, and T2DM were statistically significant between never smokers, cigarette only smokers, and dual users (Table 1). One-way ANOVA showed significant differences in the means of NAFLD scores between never smokers, cigarette only smokers, and dual users (p<0.001 for all comparisons) (Table 1). Moreover, the geometric mean of urine cotinine tended to increase across smoking types, with the highest value observed in dual users (1121.84 ng/mL, 95% CI: 1007.85–1248.73).

**Table 1:** Baseline characteristics of study participants by self-reported smoking type, among adult men from KNHANES VII-VIII (2016–2020) (N=7096)

*Characteristics*	*Smoking type*			p*
	*Never smoker (N=3110) n (%)*	*Cigarette only smoker (N=3628) n (%)*	*Dual user (N=358) n (%)*	
**Age** (years)				<0.001
20–49	1867 (60.03)	1961 (54.05)	301 (84.08)	
≥50	1243 (39.97)	1667 (45.95)	57 (15.92)	
**BMI** (kg/m^2^)				
<23.0	1172 (37.68)	1467 (40.44)	102 (28.49)	<0.001
≥23.0	1938 (62.32)	2161 (59.56)	256 (71.51)	
**Household income**				
Lower half	1129 (36.30)	1456 (40.13)	112 (31.28)	<0.001
Upper half	1981 (63.70)	2172 (59.87)	246 (68.72)	
**Education level** (years)				
≤9	518 (16.66)	772 (21.28)	21 (5.87)	<0.001
>9	2592 (83.34)	2856 (78.72)	337 (94.13)	
**Employment**				
Unemployed	921 (29.61)	791 (21.80)	61 (17.04)	<0.001
Employed	2189 (70.39)	2837 (78.20)	297 (82.96)	
**Alcohol consumption** (times/week)				
≤1	2251 (72.38)	1885 (51.96)	209 (58.38)	<0.001
>1	595 (19.13)	1658 (45.70)	144 (40.22)	
Non-response/Unknown	264 (8.49)	85 (2.34)	5 (1.40)	
**Physical activity^a^**				
Inadequate	1461 (46.98)	2086 (57.50)	166 (46.37)	<0.001
Adequate	1649 (53.02)	1542 (42.50)	192 (53.63)	
**Hypertension**				
No	2509 (80.68)	2807 (77.37)	314 (87.71)	<0.001
Yes	601 (19.32)	821 (22.63)	44 (12.29)	
**Dyslipidemia**				
No	2779 (89.36)	3052 (84.12)	319 (89.11)	<0.001
Yes	331 (10.64)	576 (15.88)	39 (10.89)	
**Type 2 DM**				
No	2896 (93.12)	3254 (89.69)	341 (95.25)	<0.001
Yes	214 (6.88)	374 (10.31)	17 (4.75)	
**HSI**, mean ± SE	33.42 ± 5.89	33.18 ± 5.80	35.31 ± 6.47	<0.001
**NRS**, mean ± SE	-2.56 ± 6.64	0.58 ± 9.77	0.58 ± 9.60	<0.001
**KNS**, mean ± SE	-1.41 ± 3.35	-0.79 ± 3.64	-0.35 ± 4.28	<0.001
	***N=1629***	***N=3492***	***N=342***	
Urine cotinine (ng/mL), geometric mean (95% CI)^b^	0.76 (0.71–0.81)	966.56 (931.31–1003.14)	1121.84 (1007.85–1248.73)	

* Derived from chi-squared tests for categorical variables and one-way ANOVA for continuous variables.

a Adequate physical activity was defined as moderate intensity activity for more than 150 min/week, high-intensity activity for more than 75 min/week, or a combination of both.

b Urine cotinine levels are expressed as geometric mean (95% CI) because they do not follow a normal distribution.

DM: diabetes mellitus. HSI: hepatic steatosis index. NRS: non-alcoholic fatty liver disease ridge score. KNS: Korea National Health and Nutrition Examination Survey non-alcoholic fatty liver disease score. SE: standard error.

Table 2 displays the results of multivariate logistic regression analysis of NAFLD status by smoking type. In the fully adjusted model, cigarette only smokers showed significantly higher odds of NAFLD relative to never smokers for all three of the NAFLD indices (HSI: AOR=1.22; 95% CI: 1.05–1.42, p=0.008; NRS: AOR=2.13; 95% CI: 1.87–2.42, p=0.000; KNS: AOR=1.33; 95% CI: 1.14–1.55, p=0.000). Dual users also had significantly higher odds of NAFLD compared to never smokers for all three NAFLD indices (HSI: AOR=1.47; 95% CI: 1.08–1.99, p=0.014; NRS: AOR=2.21; 95% CI: 1.70–2.86, p=0.000; KNS: AOR=1.35; 95% CI: 1.01–1.81, p=0.045). For all three NAFLD indices, the AORs showed an increasing pattern from never smokers to cigarette only smokers to dual users (Table 2).

**Table 2:** Multivariate logistic regression analysis of association between smoking types and NAFLD status defined by HSI, NRS, and KNS

	*Never smoker (Ref.) AOR (95% CI)*	*Cigarette only smoker AOR (95% CI)*	*Dual user AOR (95% CI)*
**HSI** (N=2046) n (%)	871 (42.57)	1026 (50.15)	149 (7.28)
Model 1	1	1.03 (0.91–1.16)	1.63 (1.26–2.11)***
Model 2	1	1.00 (0.88–1.13)	1.58 (1.22–2.04)***
Model 3	1	1.22 (1.05–1.42)**	1.47 (1.08–1.99)*
**NRS** (N=2258) n (%)	702 (31.09)	1412 (62.53)	144 (6.38)
Model 1	1	2.05 (1.83–2.30)***	2.40 (1.87–3.09)***
Model 2	1	2.03 (1.80–2.28)***	2.37 (1.84–3.04)***
Model 3	1	2.13 (1.87–2.42)***	2.21 (1.70–2.86)***
**KNS** (N=1468) n (%)	554 (37.74)	819 (55.79)	95 (6.47)
Model 1	1	1.35 (1.17–1.55)***	1.63 (1.24–2.15)**
Model 2	1	1.31 (1.14–1.51)***	1.58 (1.19–2.08)**
Model 3	1	1.33 (1.14–1.55)***	1.35 (1.01–1.81)*

Cut-off values for non-alcoholic fatty liver disease were defined as: HSI >36; NRS>0.44; KNS>0.884. Model 1: Adjusted for sociodemographic characteristics (age, income, education level, occupation). Model 2: Adjusted for Model 1 plus hypertension, type 2 diabetes mellitus, and dyslipidemia. Model 3: Adjusted for Model 2 plus body mass index, alcohol consumption, and physical activity. HSI: hepatic steatosis index. NRS: Non-alcoholic fatty liver disease ridge score. KNS: Korea National Health and Nutrition Examination Survey non-alcoholic fatty liver disease score. N = number of NAFLD events.

* p<0.05,

** p<0.01,

*** p<0.001.

Table 3 shows the subgroup analyses on the association of NAFLD, defined by HSI, by smoking type. The increasing trend for the AOR of NAFLD was maintained, although statistical significance was attenuated for the association between dual use and NAFLD for all age groups (20–49 years: AOR=1.38; 95% CI: 0.99–1.93, p=0.059; ≥50 years: AOR=2.07; 95% CI: 0.94–4.59, p=0.072), those who were overweight or obese (AOR=1.34; 95% CI: 0.98–1.83, p=0.065), all categories of alcohol consumption (≤1 time/week: AOR=1.50; 95% CI: 0.99–2.28, p=0.054; >1 time/week: AOR=1.39; 95% CI: 0.87–2.22, p=0.165), those who reported upper half of income (AOR=1.27; 95% CI: 0.88–1.82, p=0.197), those who had adequate physical activity (AOR=1.38; 95% CI: 0.89–2.15, p=0.148), and those who were diagnosed with T2DM (AOR=1.07; 95% CI: 0.39–2.96, p=0.889). No significant interaction effects were found based on age (p for interaction=0.53), BMI (p for interaction=0.20), alcohol consumption (p for interaction=0.94), income (p for interaction=0.16), physical activity (p for interaction=0.68), and diagnosis of T2DM (p for interaction=0.79).

**Table 3:** Multivariate logistic regression analysis of association between smoking types and NAFLD status, defined by HSI >36, stratified by independent variables

	*Never smoker (Ref.) AOR (95% CI)*	*Cigarette only smoker AOR (95% CI)*	*Dual user AOR (95% CI)*	*p for interaction*
**Age** (years)				0.53
20–49	1	1.12 (0.93–1.35)	1.38 (0.99–1.93)	
≥50	1	1.28 (1.00–1.65)	2.07 (0.94–4.59)	
**BMI** (kg/m^2^)				0.20
<23.0	1	1.20 (0.73–1.97)	3.18 (1.37–7.40)**	
≥23.0	1	1.22 (1.04–1.43)*	1.34 (0.98–1.83)	
**Alcohol consumption** (times/week)				0.94
≤1	1	1.21 (1.01–1.45)*	1.50 (0.99–2.28)	
>1	1	1.27 (0.96–1.69)	1.39 (0.87–2.22)	
**Income**				0.16
Lower half	1	1.21 (0.92–1.58)	2.26 (1.27–4.00)**	
Upper half	1	1.22 (1.02–1.46)*	1.27 (0.88–1.82)	
**Physical activity^a^**				0.68
Inadequate	1	1.24 (1.00–1.54)*	1.61 (1.06–2.45)*	
Adequate	1	1.22 (0.99–1.51)	1.38 (0.89–2.15)	
**Type 2 DM**				0.79
No	1	1.22 (1.04–1.42)*	1.47 (1.07–2.02)*	
Yes	1	0.94 (0.59–1.51)	1.07 (0.39–2.96)	

Cut-off value for non-alcoholic fatty liver disease was defined as: HSI >36.

a Adequate physical activity was defined as moderate intensity activity for more than 150 min/week, high-intensity activity for more than 75 min/week, or a combination of both.

DM: diabetes mellitus. HSI: hepatic steatosis index. NAFLD: non-alcoholic fatty liver disease.

* p<0.05,

** p<0.01.

Supplementary file Table S2 shows the differences in the means of log-transformed urine cotinine and pack-years of smoking between cigarette only smokers and dual users. In comparison to cigarette only smokers, dual users had significantly higher urine cotinine levels (cigarette only: 6.87; 95% CI: 6.84–6.91; dual use: 7.02; 95% CI: 6.92–7.13, p=0.02) but reported significantly lower pack-years of cigarette smoking (cigarette only: 412.17; 95% CI: 402.23–422.11; dual use: 283.39; 95% CI: 256.66–310.12, p<0.001).

Supplementary file Table S3 shows the differences in pack-years between cigarette only smokers and dual users by age group. In those aged <50 years, we observe a significant difference in pack-years between cigarette only smokers and dual users (cigarette only: 259.56; 95% CI: 250.63–268.48; dual use: 227.10; 95% CI: 205.84–248.36, p=0.0059). In contrast, in those ≥50 years, we found no significant difference in pack-years between the two groups (cigarette only: 592.02; 95% CI: 577.18–606.86; dual use: 580.65; 95% CI: 486.26–675.04, p=0.79).

Supplementary file Table S4 shows the results of a sensitivity analysis based on a multivariate logistic model adjusted for log-transformed urine cotinine in addition to the other covariates. Both cigarette only smoking (AOR=1.92; 95% CI: 1.22–3.01, p=0.005) and dual use (AOR=1.96; 95% CI: 1.18–3.25, p=0.010) were significantly associated with NAFLD defined by NRS. However, neither cigarette only smoking nor dual use was significantly associated with NAFLD when it was defined by HSI (cigarette only: AOR=1.00; 95% CI: 0.60–1.65, p=0.988; dual use: AOR=1.20; 95% CI: 0.67–2.13, p=0.540) or KNS (cigarette only: AOR=1.41; 95% CI: 0.87–2.27, p=0.161; dual use: AOR=1.46; 95% CI: 0.84–2.52, p=0.177).

## DISCUSSION

This nationally representative cross-sectional study of Korean men suggests a significant association between the dual use of combustible cigarettes and e-cigarettes and NAFLD. In multivariate logistic regression analysis, a significant association was found between dual use and NAFLD as defined by three NAFLD indices (HSI, NRS, KNS). We also observed a tendency for AORs for NAFLD to show an increasing pattern from never smokers to cigarette only smokers to dual users. To our knowledge, no other study has previously explored the association between dual use and the risk of NAFLD in Korean men by using scoring systems.

The mechanism by which smoking leads to increased risk of NAFLD is not yet clear. Animal studies have proposed that smoking modulates key proteins involved in hepatic lipogenesis, such as adenosine-5-monophosphate-activated protein kinase (AMPK) and sterol response element binding protein 1c (SREBP1c)^20,21^. In addition, longitudinal prospective studies of humans reported a significant association between smoking and NAFLD after adjustment for potential confounders, such as physical activity, alcohol consumption, diet, and body mass index (BMI)^22-24^. Large population-based studies with liver biopsies have demonstrated the association between fibrosis progression and smoking in NAFLD patients that may be modulated by insulin resistance^25,26^.

Smoking is proposed to act on the liver via three pathways: toxic, immunologic and oncogenic^5,27^. The direct toxicity of various substances in cigarettes has been reported to cause cellular injury and activate fibrosis^5^. Smoking may also cause indirect cellular injury by increasing the levels of proinflammatory cytokines and catabolic iron levels, leading to greater oxidative stress^5,28^. Smoking may also modulate the immune system in such a way that protective mechanisms are disabled while pathogenic responses are augmented^5,29^. Finally, various substances contained in cigarettes have been shown to have carcinogenic potential^5^. These pathways may be implicated in the development and progression of NAFLD^5^.

Our results suggest that dual users have higher odds of NAFLD compared to not only never smokers, but also cigarette only smokers. The reason behind this finding remains to be elucidated. It may be that dual users, who were found to have significantly higher urine cotinine levels compared to cigarette only smokers in our supplementary analysis, have greater exposure to nicotine, which was shown in prior experimental studies to aggravate hepatic steatosis. For instance, mice given a high fat diet (HFD) and regular nicotine injections showed increased hepatic steatosis compared to mice given a HFD alone^30^. In another study, ApoE knockout mice on a HFD exposed to nicotine-containing aerosol that produced serum cotinine levels similar to those of heavy smokers demonstrated increased hepatic lipid accumulation compared to control mice exposed to saline aerosol^31^.

Moreover, components of e-liquids other than nicotine, such as glycerol, may exert a gender-dependent effect on hepatic steatosis. Propylene glycol and glycerol are the main ingredients of vaping liquids, to which nicotine and flavoring are added^32^. In an experimental study, female mice, but not male mice, displayed elevated hepatic triglyceride and phosphatidylcholine levels after exposure to e-vapor composed only of glycerol (without nicotine or flavoring)^32^. Also, pregnant female mice and offspring that were exposed to nicotine-free e-vapor showed liver damage and metabolic changes^33^. These studies suggest that the components of e-liquids other than nicotine may drive the development of fatty liver.

NAFLD shows strong associations with metabolic disorders, such obesity, insulin resistance, T2DM, metabolic syndrome, and cardiovascular diseases^7^. These metabolic diseases may share risk factors and causal pathways with NAFLD^9^. In epidemiological studies, NAFLD patients were found to have a high prevalence of metabolic disorders as comorbidities^7,10^. The prevalence of comorbidities in NASH patients was determined to be 82% for obesity, 72% for hyperlipidemia, 71% for metabolic syndrome, 68% for hypertension, and 44% for T2DM in a single meta-analysis^6^. Moreover, studies have consistently reported an interactive relationship between NAFLD and T2DM and metabolic syndrome, in which the diagnosis of a disease increases the risk of having the other^26,34-36^. Moreover, while obesity is not a necessary criterion for NAFLD, it is nonetheless an important risk factor^7^. According to a longitudinal cohort study using Korea National Health Insurance Service claims data, the risk of NAFLD was significantly increased among BMI gain groups of never smokers, ex-smokers, relapsed smokers, and sustained smokers^37^. This study showed that even with smoking cessation, participants exhibited increased NAFLD risk with weight gain^37^.

NAFLD scoring systems were developed as alternatives to biopsy to detect and evaluate NAFLD^7^. Scoring systems incorporate easily measured parameters into algorithms that can be used to predict patient outcome, disease severity, or response to intervention^38^. The parameters contained in these scores are deliberately selected because they represent the factors that are closely associated with the outcome of interest^39^. The NAFLD scores used in this study reflect the different contributions of factors such as insulin resistance, hypertension, dyslipidemia, and obesity to the development of NAFLD. Also, the slightly variable findings obtained with each score may indicate that smoking impacts the development of NAFLD from multiple different physiological paths. While we cannot infer the exact biological processes or causal mechanisms underlying disease development from these scoring systems, they nevertheless provide valuable insights into the factors that are key to understanding disease pathogenesis^38,39^.

In subgroup analyses, the trend for the adjusted odds ratios of NAFLD to show an increasing pattern from never smokers to dual users was maintained, although statistical significance was attenuated in many cases after stratification by age, BMI, alcohol consumption, income, physical activity, and T2DM. No significant interaction effects were found for any of these variables in our model.

Moreover, our supplementary analyses show that dual users have significantly higher levels of urine cotinine but fewer pack-years of smoking than cigarette only smokers. The finding regarding pack-years is most likely due to the age differences between cigarette only smokers and dual users. Dual users tend to be younger than cigarette only smokers, which may explain the fewer pack-years for the former group. Indeed, the difference in mean pack-years between cigarette only smokers and dual users disappeared in individuals aged ≥50 years but was maintained in those aged <50 years. This suggests that age acts as a confounder that distorts the association between dual use and pack-years.

### Limitations

Limitations of our study include its cross-sectional design, which constrains us from assessing the temporal relationship between smoking type and NAFLD and from determining absolute risk. Moreover, while KNHANES VIII (2019–2020) differentiates between NVPs and HTPs in its survey questions, KNHANES VII (2016–2018) only asks about the use of ‘e-cigarettes’ in general. Thus, we were not able to distinguish between NVP and HTP use in this study or ascertain their individual associations with NAFLD status, despite important differences (i.e. the containment of tobacco in HTPs but not in NVPs)^16,40^. Furthermore, data on sociodemographic and health-related variables were collected through self-report and may not be accurate. Also, we were not able to exclude those who recently used nicotine replacement therapy (NRT), which may affect urine cotinine levels, due to the lack of data regarding NRT use in KNHANES VIII (2019–2020). Moreover, we were not able to use imaging data or biopsy results to determine NAFLD status and had to rely on calculated index scores. Lastly, we were unable to determine the association between e-cigarette only users and NAFLD due to insufficient sample size of the individual categories.

Strengths of our study include the fact that it is a large, population-based analysis that used reliable, nationwide data. We used strict exclusion criteria in our study and controlled for a variety of sociodemographic and health-related covariates. Also, we conducted our analysis with three different NAFLD indices, two of which were previously validated^11-13^. Our study provides the groundwork for future prospective studies to further investigate the effect of e-cigarette use on NAFLD.

## CONCLUSIONS

In this cross-sectional study of Korean men, the potential association of the dual use of combustible cigarettes and e-cigarettes with NAFLD was explored. Our results suggest that a significant association may exist between dual use and NAFLD. We observed a trend for adjusted odds ratios to increase from never smokers to cigarette only smokers to dual users, with the highest odds found in dual users.

While we evaluated the association between dual use and NAFLD indices, many questions remain. Future studies should investigate the role of additional factors that may mediate the relationship between dual use and NAFLD as well as the individual associations between NVP/HTPs and hepatic steatosis.

## Data Availability

The data supporting this research are available from the following source: https://knhanes.kdca.go.kr/knhanes/sub03/sub03_02_05.do
