# Supplementary material for: Association between the dual use of electronic and conventional cigarettes and NAFLD status in Korean men
Source: Tob Induc Dis. 2023 Feb 24;21:31. doi: 10.18332/tid/159167 (PMC9951190; doi:10.18332/tid/159167)
Supplement: Supplementary file 1 [file TID-21-31-s1.pdf]

Supplementary Table 1. Definitions of NAFLD indices

Models	Cut-off points	Equations
**Hepatic Steatosis Index (HSI)**	<30.0, >36.0	$8 \times \frac{ALT}{AST}\ ratio + BMI\ (+2, if\ diabetes;\ +2, if\ female)$
**NAFLD Ridge Score (NRS)**	<0.24, >0.44	$-0.614 + 0.007 * ALT - 0.214 * HDL + 0.053 * Triglyceride + 0.144 * HbA1c + 0.032 * WBC + 0.132 * hypertension$
**Korea National Health and Nutrition Examination Survey NAFLD score (KNS)**	<-3.285, >0.884	$0.913 \times sex(1, if\ male) + 0.089 \times WC + 0.032 \times (systolic\ blood\ pressure + fasting\ glucose) + triglycerides \times 0.007 + ALT \times 0.105 - 20.929$

Acronyms: AST, aspartate aminotransferase. ALT, alanine transaminase. BMI, body mass index. HbA1c, hemoglobin A1c. HDL, high-density lipoprotein. WBC, white blood cell count. WC, waist circumference.

Supplementary Table 2. Difference in the means of log-transformed urine cotinine and of pack-years between cigarette only smokers and dual users.

	**Cigarette only smoker**	**Dual user**	***P* value**
Urine cotinine, ng/mL	N = 3490	N = 342	
	6.87 (6.84, 6.91)	7.02 (6.92, 7.13)	0.02
Pack-years	N = 3625	N = 358	
	412.17 (402.23, 422.11)	283.39 (256.66, 310.12)	< 0.001

Urine cotinine levels are expressed as log-transformed values because they do not follow a normal distribution. Only a subsample of participants was assessed for urine cotinine.

Data are mean (95% confidence interval). *P* values are from independent samples t-tests.

Supplementary Table 3. Difference in pack-years between cigarette only smokers and dual users by age group.

Age (years)	Cigarette only smoker	Dual user	*P* value
20-49	N = 1961	N = 301	
	259.56 (250.63, 268.48)	227.10 (205.84, 248.36)	0.0059
≥50	N = 1664	N = 57	
	592.02 (577.18, 606.86)	580.65 (486.26, 675.04)	0.79

Data are pack-years (95% confidence interval). *P* values are from two-sample t-tests.

Supplementary Table 4. Association between smoking types and NAFLD status (defined by HSI, NRS, and KNS) in a multivariate logistic model adjusted for all covariates, including log-transformed urine cotinine

	**Never smoker**	**Cigarette only smoker**	**Dual user**
HSI	1.00 (reference)	1.00 (0.60, 1.65)	1.20 (0.67, 2.13)
NRS	1.00 (reference)	1.92 (1.22, 3.01)**	1.96 (1.18, 3.25)*
KNS	1.00 (reference)	1.41 (0.87, 2.27)	1.46 (0.84, 2.52)

Data are adjusted odds ratio (95% confidence interval) calculated using multivariate logistic regression analysis. Never smoker group was set as reference in calculating *P* values.
Model was adjusted for sociodemographic characteristics (age, income, education, employment status), hypertension, dyslipidemia, type 2 diabetes mellitus, body mass index, alcohol consumption, physical activity, and log-transformed urine cotinine.
*$P<0.05$; **$P<0.01$; ***$P<0.001$.
Acronyms: HSI, hepatic steatosis index; NRS, Nonalcoholic fatty liver disease ridge score; KNS, Korea National Health and Nutrition Examination Survey nonalcoholic fatty liver disease score.

© 2023 Han M. et al.
